# Implant macro-design and residual bone height on implant stability in sinus floor elevation

**DOI:** 10.6026/973206300210040

**Published:** 2025-01-31

**Authors:** Soumya Allurkar, Ali Saleh Alqahtani, Vinod Sargaiyan, Debapriya Kundu, Shagun Malik, Satya Prakash Gupta

**Affiliations:** 1 Department Oral and Maxillofacial Surgery, PMNM Dental College and Hospital, Bagalkot, Karnataka, India; 2 Department of Prosthetic Dental Science, Najran University, Najran 55461, Saudi Arabia; 3 Department of Oral Pathology & Microbiology, Maharana Pratap College of Dentistry & Research Centre, Gwalior, Madhya Pradesh, India; 4 Intern, Kalinga Institute of Dental Sciences, KIIT Deemed to be University, Patia 751024, Odhisa, India; 5 Department of Periodontology, SGT University, Gurugram, Haryana, India; 6 Department of Oral and Maxillofacial surgery, Chandra Dental College, Safedabad, Barabanki, Lucknow, Uttar Pradesh, India

**Keywords:** Sinus floor elevation, implant stability, implant macro-design, residual bone

## Abstract

The role of residual bone height (RBH) and implant macro-design on implant stability during implant placement in sinus floor evaluation (SFE) is of interest. There were a total of 160 fresh bovine rib specimens resembling type-IV density which were divided into 4 categories on the basis of residual bone height prepared **i.e*.* 3mm, 6mm, 9mm and 15mm. Implant stability quotient (ISQ) values in each of the four implant macro-design evaluated were found to increase as the height of residual bone increased. It was observed that at residual bone height (6mm, 9mm) and ISQ value were high for tapered effect design in comparison to other macro-design (p< 0.01). Thus, implant stability is affected by residual bone height and implant macro-design in maxillary sinus floor elevation technique.

## Background:

The enlargement of the maxillary antrum and resorption of alveolar ridge post extraction can impair the amount of bone in the posterior edentulous maxilla [1, 2- 3]. In order to increase the amount of bone and enable implant placement, methods for internal bone enhancement of the floor of maxillary antrum have been developed [4, 5- 6]. Growth factors and bone substitutes are utilized to prevent donor site morbidity that comes with autologous bone grafts, however bone regeneration after elevation of membrane of maxillary sinus has also been observed without application of any graft material [7, 8- 9]. Transcrestal methods for indirect elevation of maxillary sinus membrane have been devised to lessen surgical incursion and subsequent complications brought on by the formation of the bone aperture in the lateral wall of sinus [10, 11-12]. The surgical method for raising the vertical bone height for placement of implant in the atrophied maxilla is sinus floor elevation (SFE). There are two known surgical methods for SFE: "the crestal osteotome technique" and the "lateral window technique" [8, 9-10]. According to earlier research, both methods were clinically trustworthy because of the substantial rate of success following SFE [13, 14, 15-16]. The existing height of bone between the crest of maxillary bone and floor of maxillary sinus, as well as the timing of placement of implants, are some factors that must be taken into account when deciding on surgical methods [9, 10-11]. Dental implant is regarded as a very dependable and predictable therapeutic option with excellent success as well as survival rates for individuals with partial or total edentulism [7, 8-9].

The implant stability (IS) is crucial to the effectiveness of implant therapy [10, 11-12]. The area of the jaw at which the implant is positioned, surgical method, occlusal load, kind of implant and additional elements pertaining to the patient's health and lifestyle are additional considerations for success of dental implants [13, 16]. When placing implants, achieving PS is crucial. There are several elements that affect it, but the most important ones are the surgical method, implant design and bone quality [17, 18- 19]. Primary stability is influenced by several factors, including as bone density, surgical technique and implant design. Most importantly, selecting an implant design can be a dependable method of boosting primary stability [20, 21-22]. Even though some implant designs have shown improved stability in high bone density, primary stability can drastically decrease in low-density bone. In order to achieve primary stability in low-density bone, suitable implant designs must be considered [23- 24]. It has been suggested that specific implant macro-designs, such as implant form, pitch, depth, thickness and thread face angle, positively affect primary stability. Many implant designs have been developed and are currently available for purchase [25-26]. Therefore, it is of interest to investigate the impact of residual bone height and implant macro-design on primary stability of implants placed in the atrophic maxillary sinus floor after sinus floor elevation.

## Methods and Materials:

## Specimens for an SFE simulated model (an *ex vivo* model):

Computerized tomography (CT) was used to confirm that the fresh bovine rib samples resembled type-IV density. There were total 160 specimens. They were divided into 4 categories on the basis of residual bone height prepared *i.e* 3mm, 6mm, 9 mm and 15 mm. In each category of specimens with specific RBH, 40 implants belonging four macro-designs were inserted (10 implant of one macro-design) (Table 1). Four macro-designs of implants were selected. They were "Straumann® Standard Plus implants (SP; length 10 mm, diameter 4.1 mm)", "Straumann® Tapered Effect implants (TE; length 10 mm, diameter 4.1 mm)", "Straumann® Bone Level implants (BL; length 10 mm, diameter 4.1 mm)", "Straumann® Bone Level Tapered implants (BLT; length 10 mm, diameter 4.1 mm)" Bone blocks (about 50 mm x 20 mm x 5 mm) were made from fresh bovine rib using a round bur and a water-cooled precision diamond saw (YSC-500FDX, Yutaka, Aichi, Japan). The external surfaces of the bone blocks were carefully cleansed by rinsing in water after the surrounding soft tissue was removed. Every block was examined macroscopically for anomalies and a precision caliper was used to confirm the thickness (3mm, 6mm, 9 mm and 15mm). The purpose of this thickness was to mimic the existent atrophic maxillary bone.

## Implant placement in an SFE simulated model:

The implants were inserted into bone blocks that were 3 mm, 6 mm, 9 mm and 15 mm thick (a SFE generated model). The implant placements were done by two people. Placement was done in a random order. Following the manufacturer's standard insertion protocol, all implants were placed from the cutting segments, which were made entirely of cancellous bone"(Straumann®, Institute Straumann AG and Basel, Switzerland)". The digital torque driver was used to capture the maximum insertion torque (MIT) values at implant insertion "(CEDAR, Sugisaki Keiki and Ibaraki, Japan)".

## Implant stability quotient (ISQ) value:

The "Osstell TM Mentor resonance frequency analysis transducer (Model 6.0, Integration Diagnostics, Göteborg, Sweden)" was used to measure the resonance frequencies of bones. It was used to measure ISQ values. The transducers were manually screwed into place on the implants. Following the manufacturer's instructions, the system's frequency response was recorded and measurements were taken from the left, right, front and rear.

## Statistical analysis:

Statistical analyses were conducted using SPSS software (version 16.0, SPSS Inc., Chicago, IL, USA). After one-way ANOVA, the Tukey test was used to establish the statistical significance of the group differences, with p values less than 0.05 being deemed significant.

## Results:

The ISQ values in each macro-design were found to increase as the height of residual bone increased. The ISQ values were maximum at 15 mm residual bone height followed by 9mm, 6mm and 3mm. The findings were significant statistically (p< 0.01). It was observed that at residual bone height (6mm, 9mm), ISQ values were maximum for tapered effect design in comparison to other macro-design (p< 0.01). It was also observed that the difference in ISQ values between different macro-design at 3 mm residual bone height and 15 mm residual bone height was non-significant statistically. These findings showed that implant stability is significantly associated with residual bone height and implant macro-design (Table 2).

## Discussion:

A number of variables, including as implant design, surgical technique, bone density and influence primary stability. Above all, choosing an implant design can be a reliable way to increase primary stability [10, 11, 12-13]. Primary stability can significantly decline in low-density bone, despite the fact that several implant designs have demonstrated increased stability in high bone density. In low-density bone, appropriate designs of implant needs to be taken into account in order to attain primary stability [14, 15, 16- 17]. It has been proposed that certain macro designs of implants, including shape of implant, shape, pitch, depth, thickness and face angle of thread, have a beneficial impact on primary stability [19, 20-21]. This study was conducted to investigate the impact of residual bone height and implant macro design on primary stability of implants placed in the atrophic maxillary sinus floor. The findings of this study showed that implant stability is significantly associated with residual bone height. The ISQ values in each macro-design were found to increase as the height of residual bone increased. The ISQ values were maximum at 15 mm residual bone height followed by 9mm, 6mm and 3mm. The findings were significant statistically (p< 0.01). There are some studies which have results similar to results of present study [24, 25- 26]. These studies also stated that implant stability is significantly associated with residual bone height in sinus floor elevation. These studies like our study also stated that implant stability increases as the residual bone height increases [23, 24-25]. According to earlier research, both methods were clinically trustworthy because of the substantial rate of success following SFE [20, 21, 22, 23-24]. The existing height of bone between the crest of maxillary bone and floor of maxillary sinus, as well as the timing of placement of implants, are some factors that must be taken into account when deciding on surgical methods [23, 24, 25-26]. In our study, it was observed that at residual bone height (6mm, 9mm), ISQ values were maximum for tapered effect design in comparison to other macro-design (p< 0.01). It was also observed that the difference in ISQ values between different macro-design at 3 mm residual bone height and 15 mm residual bone height was non-significant statistically. The observations of our study are supported by the results of other studies which also stated that implant macro-design can also affect the implant stability [24, 25- 26]. A study stated that cylindric implant when inserted along with sinus floor elevation can provide better implant stability at residual bone height of 6 mm and 9mm [25, 26- 27]. For people with partial or complete edentulism, dental implants are seen to be a very reliable and predictable therapeutic choice with great success and survival rates. For implant therapy to be effective, implant stability (IS) is essential [21, 22-23]. Other factors that affect the success of dental implants include the kind of implant, occlusal load, surgical technique, the location of the jaw where the implant is placed and other factors related to the patient's health and lifestyle [17, 18-19]. Achieving PS is essential with implant placement. Although a number of factors influence it, the surgical technique, implant design and bone quality are the most crucial [20, 21-22].

## Conclusion:

Data shows that the implant stability is affected by residual bone height and implant macro-design in maxillary sinus floor elevation technique.

## Figures and Tables

**Table 1:** Distribution of study specimens

	**Residual bone height**			
	**3 mm**	**6 mm**	**9 mm**	**15 mm**
**Implant macrodesign**				
Standard Plus (SP)	10	10	10	10
Tapered Effect (TE)	10	10	10	10
Bone level (BL)	10	10	10	10
Bone level tapered (BLT)	10	10	10	10

**Table 2:** ISQ values (mean ± SD) of implants with different macro-designs at different residual bone height

	**Residual bone height**				
	**3 mm**	**6 mm**	**9 mm**	**15 mm**	**P value**
**Implant macro-design**					
Standard Plus (SP)	51.17±0.63	63.06±0.52	67.15±0.41	74.24±0.31	< 0.01
Tapered Effect (TE)	53.29±0.83	70.18±0.72	74.14±0.61	77.59±0.43	< 0.01
Bone level (BL)	52.32±0.74	67.86±0.63	70.82±0.52	74.36±0.51	< 0.01
Bone level tapered (BLT)	52.24±0.04	69.30±0.96	72.41±0.87	75.11±0.17	< 0.01
P value	0.51	< 0.01	< 0.01	0.62	

## References

[1] Emmert M (2022). J Oral Implantol..

[2] Bataineh A.B, Al-Dakes A.M (2017). J Clin Exp Dent..

[3] Danesh-Sani S.A (2016). Br J Oral Maxillofac Surg..

[4] Yamaguchi Y (2020). Int J Implant Dent..

[5] Lan T.H (2012). Clin Oral Investig..

[6] Lundgren S (2017). Periodontol 2000..

[7] Lozano-Carrascal N (2016). Med Oral Patol Oral Cir Bucal..

[8] Raghoebar G.M (2019). J Clin Periodontol..

[9] Almutairi A.S (2018). F1000Res..

[10] Herrero-Climent M (2020). Int J Environ Res Public Health..

[11] Zhou Y (2021). Clin Oral Implants Res..

[12] Abuhussein H (2010). Clin Oral Implants Res..

[13] Ogino Y (2021). Materials (Basel)..

[14] Toyoshima T (2015). Clin Implant Dent Relat Res..

[15] Farina R (2022). Clin Oral Investig..

[16] Falco A (2018). Int J Oral Maxillofac Implants..

[17] Al-Dajani M (2016). Clin Implant Dent Relat Res..

[18] Romanos G.E (2014). Clin Implant Dent Relat Res..

[19] Moraschini V (2017). Int J Oral Maxillofac Surg..

[20] Romanos G.E (2014). Clin Implant Dent Relat Res..

[21] Duan D.H (2017). J Periodontol..

[22] Javed F (2013). Interv Med Appl Sci..

[23] Verdugo F (2017). Clin Implant Dent Relat Res..

[24] Molly L (2006). Clin Oral Implants Res..

[25] Imai M (2022). BMC Oral Health..

[26] Khaled H (2019). Clin Implant Dent Relat Res..

[27] Tokuc B (2023). J Oral Implantol..

